# The Expression of the Ubiquitin Ligase SIAH2 (Seven *In Absentia* Homolog 2) Is Increased in Human Lung Cancer

**DOI:** 10.1371/journal.pone.0143376

**Published:** 2015-11-18

**Authors:** Paula Moreno, Maribel Lara-Chica, Rafael Soler-Torronteras, Teresa Caro, Manuel Medina, Antonio Álvarez, Ángel Salvatierra, Eduardo Muñoz, Marco A. Calzado

**Affiliations:** 1 Department of Cell Biology, Physiology and Immunology, University of Córdoba, Spain, Instituto Maimónides de Investigación Biomédica de Córdoba (IMIBIC)/ Hospital Universitario Reina Sofía, 14004 Córdoba, Spain; 2 Department of Pathology, Hospital Universitario Reina Sofía, Spain, Instituto Maimónides de Investigación Biomédica de Córdoba (IMIBIC)/ University of Córdoba, 14004 Córdoba, Spain; 3 Thoracic Surgery and Lung Transplantation Unit, Hospital Universitario Reina Sofía, 14004 Córdoba, Spain; Cincinnati Children's Hospital Medical Center, UNITED STATES

## Abstract

**Objectives:**

Lung cancer is the leading cause of cancer-related deaths worldwide. Overall 5-year survival has shown little improvement over the last decades. Seven in absentia homolog (SIAH) proteins are E3 ubiquitin ligases that mediate proteasomal protein degradation by poly-ubiquitination. Even though SIAH proteins play a key role in several biological processes, their role in human cancer remains controversial. The aim of the study was to document SIAH2 expression pattern at different levels (mRNA, protein level and immunohistochemistry) in human non-small cell lung cancer (NSCLC) samples compared to surrounding healthy tissue from the same patient, and to analyse the association with clinicopathological features.

**Materials and Methods:**

One hundred and fifty-two samples from a patient cohort treated surgically for primary lung cancer were obtained for the study. Genic and protein expression levels of SIAH2 were analysed and compared with clinic-pathologic variables.

**Results:**

The present study is the first to analyze the SIAH2 expression pattern at different levels (RNA, protein expression and immunohistochemistry) in non-small cell lung cancer (NSCLC). We found that SIAH2 protein expression is significantly enhanced in human lung adenocarcinoma (ADC) and squamous cell lung cancer (SCC). Paradoxically, non-significant changes at RNA level were found, suggesting a post-traductional regulatory mechanism. More importantly, an increased correlation between SIAH2 expression and tumor grade was detected, suggesting that this protein could be used as a prognostic biomarker to predict lung cancer progression. Likewise, SIAH2 protein expression showed a strong positive correlation with fluorodeoxyglucose (2-deoxy-2(^18^F)fluoro-D-glucose) uptake in primary NSCLC, which may assist clinicians in stratifying patients at increased overall risk of poor survival. Additionally, we described an inverse correlation between the expression of SIAH2 and the levels of one of its substrates, the serine/threonine kinase DYRK2.

**Conclusions:**

Our results provide insight into the potential use of SIAH2 as a novel target for lung cancer treatment.

## Introduction

Lung cancer continues to be the leading cause of cancer-related mortality worldwide, accounting for nearly 1.4 million deaths annually [1]. Contrary to breast or prostate cancers, in which survival has improved significantly, overall 5-year survival for lung cancer has shown little improvement over the last two or three decades. Thus, the relative 5-year survival rate is 11–15% [2, 3], which means that 90% of patients will die of the disease.

Curative-intent pulmonary resection offers the best opportunity for cure when the tumors are localized within the lung [4]. Unfortunately, the majority of patients present with an advanced disease (stages III and IV), being systemic therapy with chemotherapy and/or radiation therapy the most beneficial treatment modality. In the last decade, several new molecular therapeutic targets have been described, but their efficiency and clinical impact remains unclear [5]. Altogether, these data highlights the need of new and more effective therapies.

Seven in abstentia homolog (SIAH) proteins are RING (Really Interesting New Gene) finger E3 ubiquitin ligases, which mediate proteasomal protein degradation by poly-ubiquitination [6]. Structurally, the SIAH family present a divergent N-terminal domain, a highly conserved catalytic RING domain, two zinc finger domains and a substrate-binding domain [7–9]. Mice express three members of the family, Siah1a, Siah1b and Siah2. Two SIAH proteins have been identified in humans, SIAH1 and SIAH2 [10, 11], which can exert distinct functions in cellular processes including cell cycle control, DNA damage response, tumorigenesis and metastasis [12]. Several SIAH substrates have been described to date, including the hypoxia-regulating family of prolyl hydroxylases (PHDs), PML, TRAF2, PPAR, AKAP121, HDAC3, DCC, HIPK2 and DYRK2 [13–15]. Consequently, SIAH proteins are key players in biological processes like DNA damage response, hypoxia pathway, estrogen signaling, inflammation, apoptosis, and tumor suppression [12, 15].

The role of SIAH proteins in human cancer remains controversial. At present, the number of studies that link the expression of SIAH with the development of human cancer is very limited, presenting contradictory evidence that classifies SIAH proteins either as an oncogene or as a tumor suppressor. On the one hand, several groups have shown an oncogenic role for SIAH proteins, especially SIAH2, in breast [16–18], prostate [19, 20] and liver cancer [21]. On the contrary, SIAH proteins (especially SIAH1) have been found to act as a tumor suppressor in breast cancer [22], gastric tumors [23] and liver cancer [24]. This could be explained as a consequence of the specificity of each subunit to degrade different substrates. Thus, we can conclude that each subunit plays a different role in the tumorigenesis control, which has been recently reviewed by Wong SF and Moller A [25].

Mainly due to the oncogenic role of SIAH2 in some types of cancer, the objectives of our current study were to document for the first time the pattern of SIAH2 expression at different levels (mRNA, protein level and immunohistochemistry) in human non-small cell lung cancer (NSCLC) samples compared with surrounding healthy tissue from the same patient, and to analyse its association with clinicopathological features. Moreover, the correlation with the expression of one SIAH2 substrate, the serine/threonine kinase DYRK2 (Dual specificity tyrosine-phosphorylation-regulated kinase 2)[26] was also explored.

The novelty of this work relies on the analysis of SIAH2 expression pattern at different levels (RNA, protein expression and immunohistochemistry) in human NSCLC compared to healthy lung from the same patient. The observed results herald the possible use of SIAH2 as a prognostic biomarker of lung cancer.

## Material and Methods

### Patients and Samples

Lung tissue samples from patients treated surgically for primary lung cancer were obtained at University Hospital Reina Sofia (Cordoba, Spain) from January 2011 to December 2012. The collection, use and storage of tissue samples was approved by the Ethics and Clinical Research Committee of the University Hospital Reina Sofia. All patients signed a written informed consent document indicating their voluntary donation. In parallel, clinicopatological data were prospectively collected, including: demographic data, comorbidities, history of neoplasms, tumor size, SUV(max) of tumor (maximum standardized uptake value, as measure of ^18^FDG uptake), pTNM, tumor grade, and development of metastases during follow-up. Tissue specimens were immediately frozen with RNALater^®^ (SIGMA, St. Louis-MO, USA) and stored at -80°C until RNA extraction and protein analysis was done. The remaining specimen was formalin-fixed and paraffin-embedded for conventional diagnostic studies and immunohistochemical analysis. All samples included in this study were histologically reviewed and classified as adenocarcinoma (n = 36) or squamous-cell lung cancer (n = 40). In addition, adjacent normal lung tissue was also taken from all patients. None of the patients had received chemotherapy or radiation therapy before the operation. Human biological samples used were collected, stored and managed by the Cordoba node belonging to the Biobank of the Andalusian Health Service (Servicio Andaluz de Salud-SAS).

### Histologic analysis

Immunohistochemical staining was performed on formalin-fixed, paraffin-embedded human lung tumors. Staining of cancer cells was scored and compared to peripheral normal lung tissue. Five-micrometer sections from tumor samples were prepared for tissue slides. Sections were deparaffinised in xylene, and rehydrated in a graded ethanol series and distilled water. Staining with hematoxylin–eosin (H & E) and the mouse SIAH mAb 24E6H3 (Novus Biologicals, Cambridge, UK) at 1:40 dilution or the rabbit polyclonal anti-DYRK2 antibody (AP7534a; Abgent, San Diego, Calif) at 1:50 dilution was performed overnight at 4°C. Antigen retrieval for sections to be stained with the anti-SIAH mAb or the polyclonal anti-DYRK2 antibody was performed by incubating the slides in 1 mM EDTA pH 8.0 in a 98°C–100°C steamer for 30 minutes. SIAH2 IHC staining was evaluated as follows: 0, no staining or faint cytoplasmic staining in less than 10% of tumor cells; 1+, faint cytoplasmic staining in more tan 10% of tumor cells; 2+, weak or moderate cytoplasmic staining in more than 10% of tumor cells; 3+, more than 10% of strong cytoplasmic staining. As reported previously, 0 or 1+ staining intensity was considered as DYRK2 negative, whereas 2+ or 3+ was considered as DYRK2 positive.

### mRNA extraction and qPCR

Total RNA was extracted from tissues stored in RNAlater^**®**^ using RNeasy Mini Kit (Qiagen) according to the manufacturer protocol, and treated with 1 μl (1unit/μl) DNAse-1 (Sigma, USA). Total RNA concentration and integrity were analyzed using Experion^**®**^ automated electrophoresis station (Bio-Rad, CA, USA). All the samples analyzed showed a RIN value (RNA Integrity Number) higher than 9. Reverse transcription was performed with the iScript cDNA Synthesis Kit (Bio-Rad). Real-time PCR was employed with GoTaq qPCR Master Mix (Promega, Madison, WI, USA) in an iCYCLER detection system (Bio-Rad). The amplification profile consisted of an initial denaturation for 5 min at 95°C and then 40 cycles of 30 s at 95°C, annealing for 10 s at a temperature of 61.4°C (SIAH2 gene) or 56°C (DYRK2 gene), 30 seconds at 72°C for extension and one denaturation step of 1 minute at 97°C. Amplification efficiencies were validated and normalized against β-actin and HPRT, and fold change in gene expression was calculated using the 2^−ΔΔCt^ method. The following primers were used:

SIAH2-forward: 5’-CTATGGAGAAGGTGGCCTCG-3’

SIAH2-reverser: 5’-CGTATGGTGCAGGGTCAGG-3’

DYRK2-forward: 5’-GTGGTCAAGGCCTACGATCACA-3’

DYRK2-reverse: 5’-CCGCAGGTGTTCCAGGATTC-3’

HPRT-forward: 5’-ATGGGAGGCCATCACATTGT-3’

HPRT-reverser: 5’-ATGTAATCCAGCAGGTCAGCA-3’

β-actin-forward: 5’-GCTCCTCCTGAGCGCAAG-3’

β-actin-reverser: 5’-CATCTGCTGGAAGGTGGACA-3’

### Western blotting and antibodies

Total protein extracts from lung cancer samples and matched adjacent normal lung were extracted from RNAlater^**®**^ preserved tissue. Membranes were first incubated overnight at 4°C with primary antibodies (anti-SIAH2: sc-5507, dilution 1:500, Santa Cruz; anti-DYRK2: AP7534a, dilution 1:500, Abgent; anti β-actin: clone C4, dilution 1:1000, Santa Cruz). Washed membranes were then incubated with appropriate secondary antibodies coupled to horseradish peroxidase. SIAH2 protein signal intensities on western blots were analyzed and quantified using ImageJ v1.45 software (http://rsbweb.nih.gov/ij/), after normalization to β-actin signal intensities.

### BEAS-2B squamous-cell differentiation model

BEAS-2B cells were purchased from SIGMA (SIGMA; European Collection of Cell Cultures, Salisbury, UK. 95102433) and maintained in serum free LHC-9 medium (Life Technologies, Carlsbad, CA, USA) at 37°C in a humidified atmosphere containing 5% CO_2_. Squamous differentiation was acquired after culturing in LHC-9 medium enriched with 10% FBS during 7 days.

### Statistical Analysis

Pearson’s chi-square test and Fisher’s exact test were used to assess differences between categorical variables. Unpaired t test was used to compare means between two quantitative variables from normally distributed data, and Mann-Whitney test for non-normally distributed data. Kruskal-Wallis test was used for variables with more than two categories. Linear regression analyses were performed to assess the relationship between two continuous variables. Continuous variables are expressed as means ± standard deviation. Categorical variables are expressed as counts and proportions. Differences with p values less than 0.05 were considered significant. The statistical analysis was performed using SPSS (SPSS 20.0 for Mac: SPSS Inc., Chicago, IL, USA).

## Results

### The mRNA expression of SIAH2 is not altered in human lung cancer

**Fig 1:**
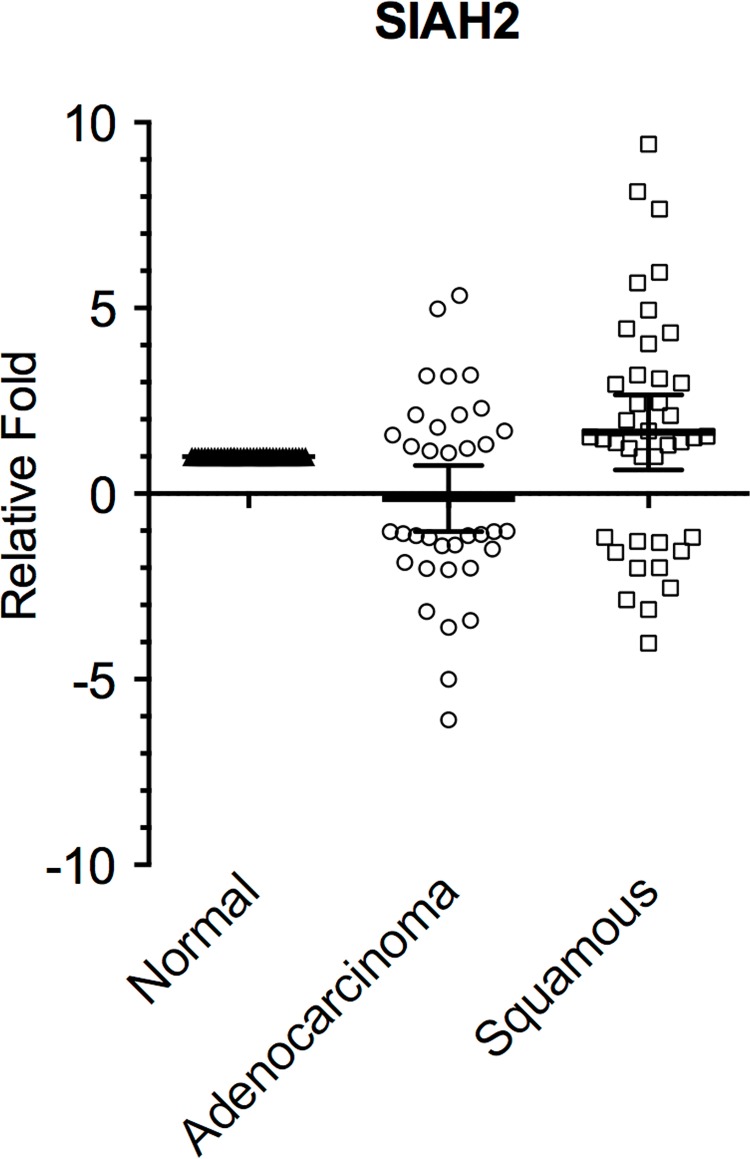
SIAH2 mRNA expression levels in NSCLC. Total RNA was extracted from tissues, integrity evaluated (all the samples included showed a RIN above 9) and changes in expression of SIAH2 in tumor samples compared to normal lung samples from the same patient analyzed by qPCR and expressed as a fold-change. Amplification efficiencies were validated and normalized against β-actin and HPRT, and fold change in gene expression was calculated using the 2^−ΔΔCt^ method. Results represent the mean ± SD.

Seventy-six surgically resected human lung cancer specimens with matched samples of adjacent normal lung tissue were examined (n = 36 ADC, n = 40 SCC). The clinicopathological characteristics of the entire patient cohort are summarized in Table 1. Changes in expression of SIAH2 in tumor samples compared to normal lung samples were expressed as a fold-change. As shown in Fig 1, subtle differences in SIAH2 gene expression were found in both ADC and SCC samples, though not significant.

**Table 1:** Patient characteristics.

Characteristics	Adenocarcinoma (n = 36)	Squamous cell (n = 40)	p-Value
**Age (Mean)**	62.22±9.83	68.79±7.46	*0*.*002*
**Sex**			*0*.*001*
*Male*	27	40	
*Female*	9	0	
**Comorbidities**	28	37	*0*.*02*
**Neoplasms**	9	2	*0*.*02*
**Metastases in follow- up**	7	0	*0*.*005*
**Tumor size**	3.62±1.96	4.6±2.06	*0*.*04*
**SUV(max)** *	11.55±8.16	13.4±5.73	*0*.*35*
**pTNM**			*0*.*479*
IA	11	6	
IB	8	13	
IIA	7	8	
IIB	4	7	
IIIA	5	6	
IIIB	1	0	
**Grade Differentiation**			*0*.*112*
I	4	2	
II	16	18	
III	11	18	
N.S.	5	2	

*Data available in 50 cases

### SIAH2 protein levels are overexpressed in human lung cancer

**Fig 2:**
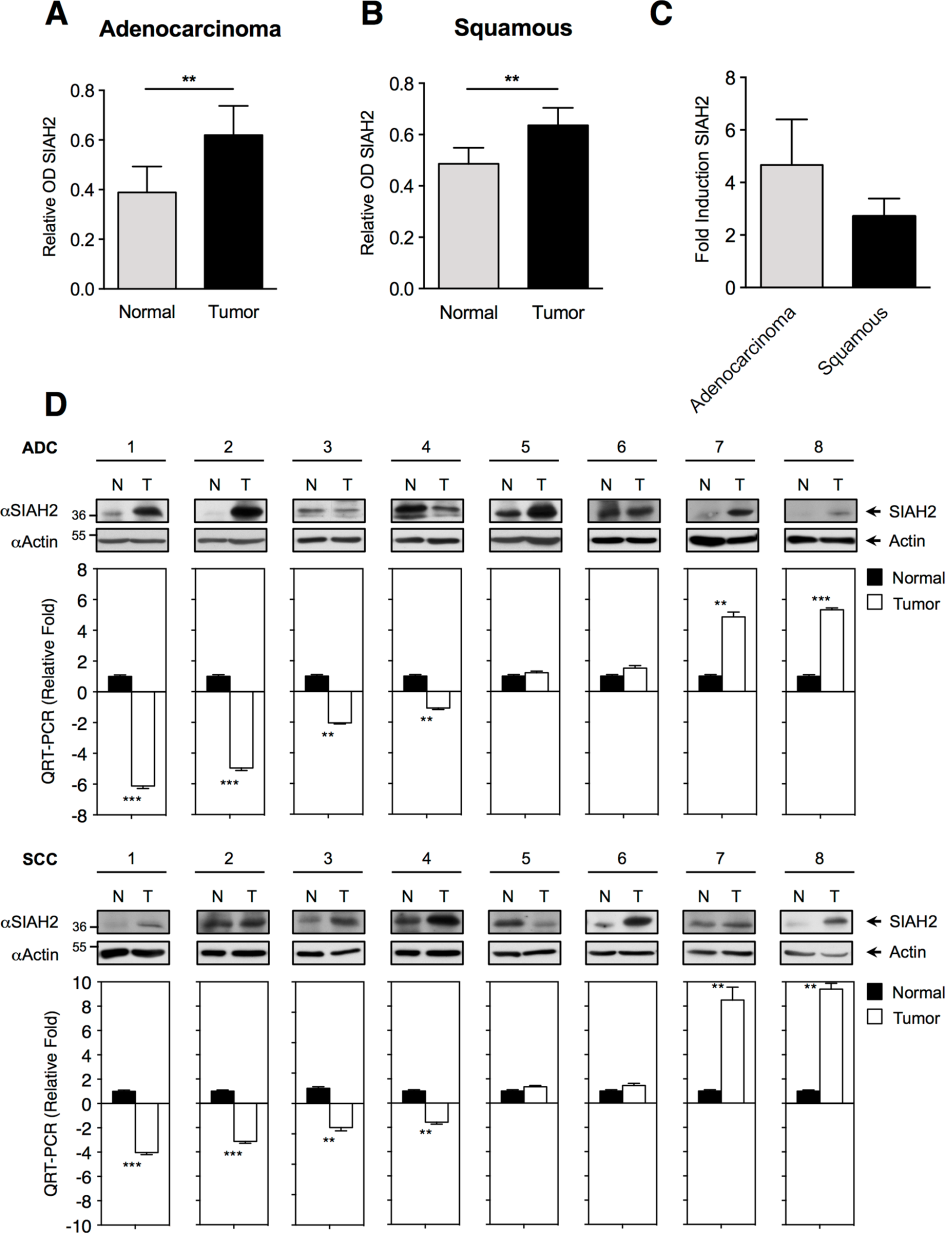
SIAH2 protein expression in human lung cancer compared to surrounding healthy tissue. (A, B) Total proteins were extracted from ADC or SDC specimens and the corresponding normal lung tissue, SIAH2 protein expression analyzed by western blot and the bands quantified by densitometry after normalization to actin signal intensities. The results are expressed as relative OD expression and represent the mean ± SD. ** *p* < 0.001. (C) Fold induction representation of SIAH2 in tumor samples compared to normal lung samples from the same patient. Results represent the mean ± SD. (D) Results from 16 patients (8 ADC and 8 SCC) selected to represent a range of differential mRNA expression between normal lung tissue (N) and tumor (T). SIAH2 protein expression was analyzed by immunoblots (upper panel) and mRNA expression by qPCR (lower panel). Data are mean ± SD of n = 3 experiments. ** *p* < 0.001 and *** *p* < 0.0001.

We next investigated whether SIAH2 protein expression is altered in human lung cancer. For this purpose, total proteins were extracted from the same 76 lung cancer specimens used above, and SIAH2 protein expression in lung cancer tissue compared to normal lung tissue was examined by Western blot. SIAH2 protein expression in lung cancer samples was compared to that of the corresponding healthy tissue after normalization to actin signal intensities, and expressed as relative OD expression (Representative gel in S1 Fig). As showed in Fig 2, we found that SIAH2 protein expression was significantly increased in both ADC and SCC specimens compared to normal lung tissue (Fig 2A and 2B). Moreover, after analyzing the fold induction of SIAH2 in tumor samples compared to normal lung samples from the same patient, the highest SIAH2 protein expression was found in ADC, in which the expression was increased by 4-fold compared to SCC, which showed a 2-fold increase in SIAH2 protein levels (Fig 2C).

Due to the difference in the induction observed between SIAH2 mRNA and protein levels, we decided to analyze in more detail a range of 16 samples (8 ADC and 8 SCC). These samples were selected to represent a wide range of SIAH2 mRNA differential expression between tumor and normal tissues. As showed in Fig 2D, in most of the cases SIAH2 protein expression is elevated in tumors irrespective of the mRNA expression. These results clearly indicate the existence of a change or alteration in the control mechanisms at post-transcriptional level in tumor cells compared to healthy tissue.

### SIAH2 expression is increased in high-grade lung carcinomas

**Fig 3:**
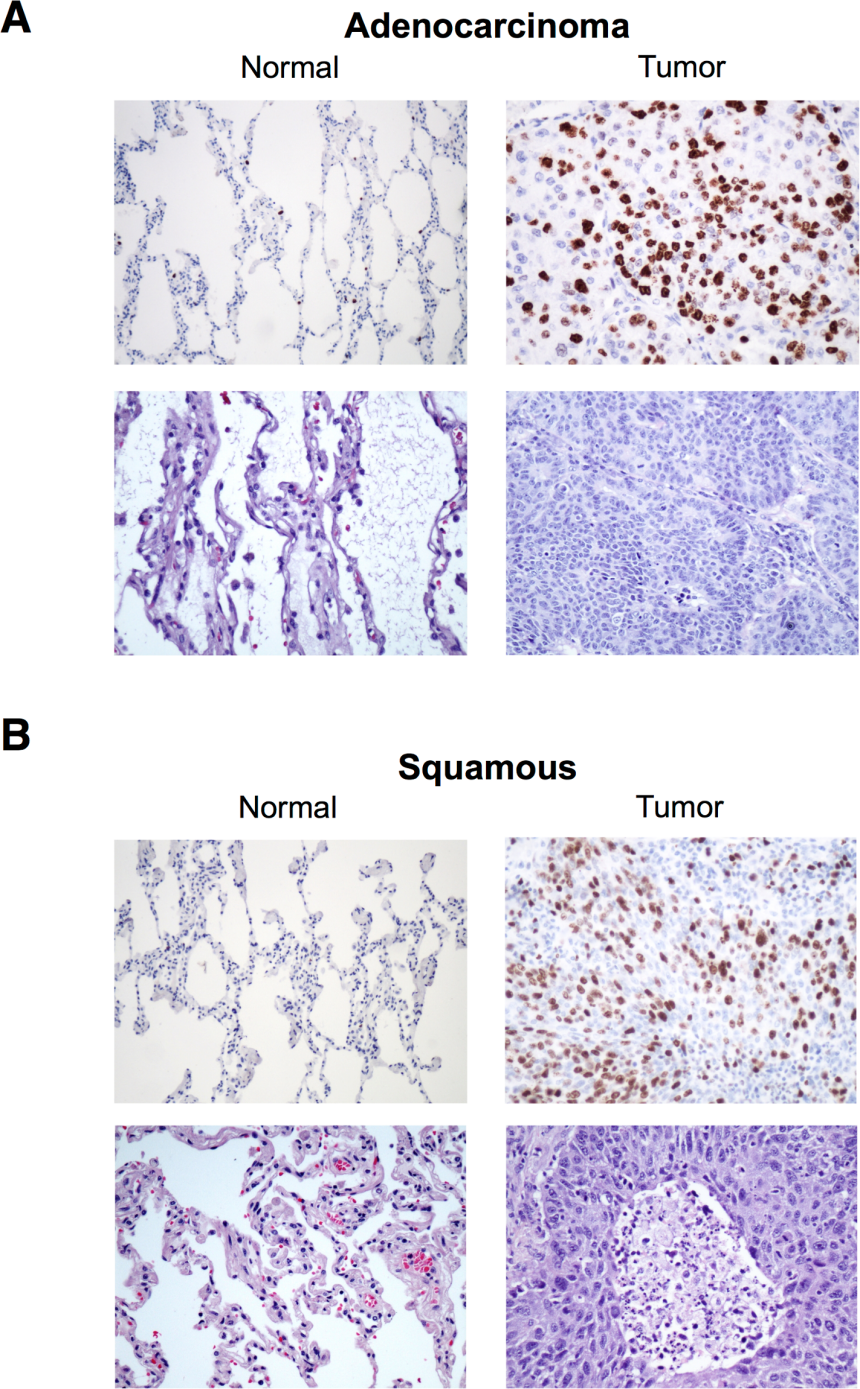
Expression of SIAH2 in ADC and SCC analyzed by immunohistochemistry. Representative images of lung tumor and adjacent normal tissue stained with SIAH2 antibody and hematoxylin–eosin (HE)(x100).

In order to test whether SIAH2 expression could be correlated with lung carcinogenesis, we decided to evaluate SIAH2 expression and its localization pattern in lung cancer by immunohistochemistry in the complete patient cohort. We observed that SIAH2 expression was highly detectable in both major types of human lung cancer, being the expression predominantly nuclear (Fig 3). Moderate to strong homogeneous staining was identified in lung cancer specimens compared to normal lung tissue, which showed isolated and occasional cellular staining.

**Fig 4:**
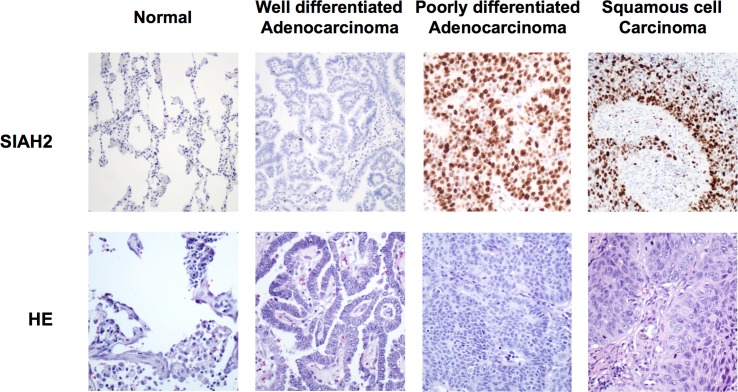
Correlation between SIAH2 expression and tumor grade. Representative images of immunohistochemical analysis of SIAH2 in normal lung, well and poorly differentiated adenocarcinoma and squamous cell carcinoma. Occasional nuclear positivity in normal lung, moderate staining in well differentiated adenocarcinoma and strong staining in poorly differentiated adenocarcinoma and squamous cell carcinoma (HE: hematoxylin–eosin)(x100).

To further examine whether endogenous SIAH2 protein expression changes with lung tumor aggressiveness, we analyzed SIAH2 expression in different human lung cancers, including well-differentiated ADC, poorly differentiated ADC and SCC. As shown in Fig 4, the percentage of tumor cells that expressed SIAH2 increased with the histological tumor grade, being highest in poorly differentiated lung cancer specimens. Moreover, a difference of SIAH2 expression between SCC and ADC was detected, with SCC being more intensively stained for SIAH2 compared to ADC.

### Association between SIAH2 expression and clinicopathological features

**Fig 5:**
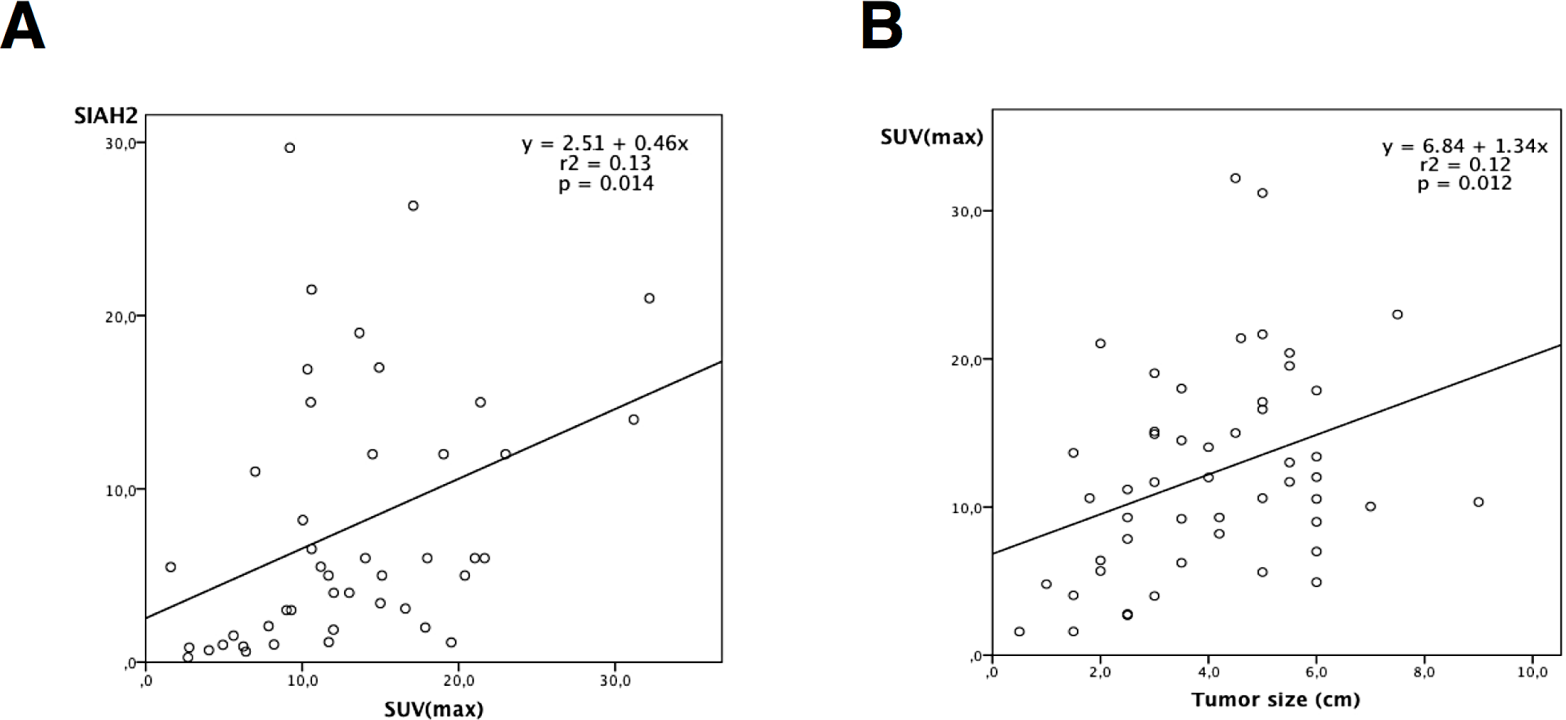
Scatter plot showing results of Pearson´s correlation analysis. (A) Positive correlation between SIAH2 protein expression and 18FDG uptake (measured as maximum standardized uptake value-SUVmax) in primary NSCLC (*p* = 0.014), r2 = 0.13). (B) Positive correlation between increasing tumor size and 18FDG uptake on PET/CT scans (*p* = 0.012, r2 = 0.12).

To evaluate the biological significance of SIAH2 expression in human lung cancer, we examined the relationship between different clinicopathological characteristics (S1 Table) and changes in SIAH2 expression levels (mRNA and protein) in tumors compared to healthy lung tissue. IHC data were not included because SIAH2 expression was positive in all tumors. The Seventh Edition of the TNM Classification for Lung Cancer (Lung Cancer Staging Project, International Association for the Study of Lung Cancer-IASLC)[27] was used for staging. It is noteworthy that SIAH2 protein expression showed a strong correlation with SUV(max) in primary NSCLC (*p* = 0.014). Moreover, increasing tumor size correlated with high uptake of ^18^FDG in PET/CT scans (*p* = 0.012) (Fig 5).

### DYRK2 protein expression is reduced in human lung cancer

**Fig 6:**
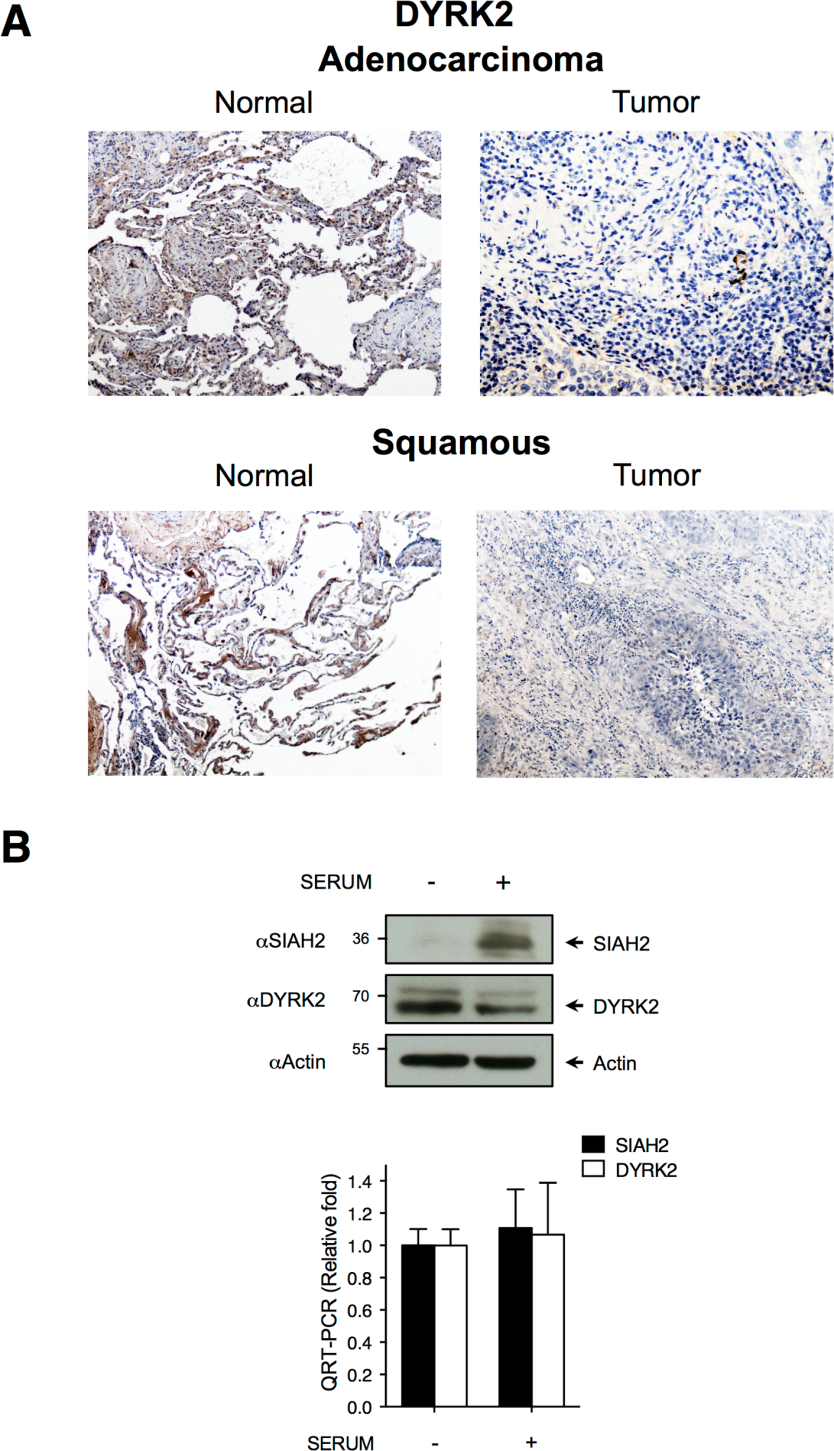
SIAH2 protein expression correlates with expression of DYRK2. (A) Representative images of adenocarcinoma or squamous cell carcinoma, and adjacent normal tissue stained with DYRK2 antibody (x100). (B) BEAS-2B cells were cultured with or without serum during 7 days, lysed and protein expression was evaluated by immunoblot with the indicated antibodies (upper panel) and mRNA expression by qPCR (lower panel). Representative blot out of three independent experiments and the positions and molecular weights (in kDa) are indicated.

Next, we decided to correlate SIAH2 expression with some of its substrates. We then analyzed the possible impact of the previously demonstrated mutual regulation between SIAH2 and DYRK2 in human lung cancer [26] by immunohistochemistry. DYRK2 plays an important role on the DNA damage-signaling pathway and its lung expression has not been deeply studied yet. DYRK2 expression was determined in the complete patient cohort and representative images are shown in Fig 6A. We observed a significant increase in DYRK2 expression in healthy lung specimens compared to corresponding lung cancer samples, suggesting that SIAH2 overexpression is associated with decreased expression of its substrates.

Finally, we decided to evaluate the aforementioned results using an *in vitro* differentiation model of progenitor cells to lung carcinoma. Therefore, we investigated SIAH2 and DYRK2 protein and mRNA expression levels in the human bronchial epithelial cell line BEAS-2B. An inverse expression between SIAH2 and DYRK2 protein expression was found, either in normal epithelial cells or when subjected to squamous differentiation (Fig 6B), without observing significant changes at the mRNA level. Hence, we observed that DYRK2 expression was significantly reduced in squamous phenotype compared with normal epithelial cells. On the contrary, SIAH2 levels increased significantly following phenotype squamous differentiation. These results using an *in vitro* differentiation model reinforce the association between SIAH2 protein expression and lung carcinoma observed in the human samples, which is also associated with a decreased expression of substrates such as DYRK2.

## Discussion

Current literature includes contradictory data with regard to the role of SIAH in cancer progression. Therefore, it is of paramount importance to study each individual form separately in order to discriminate their predominant role on the development of different neoplasms. Nevertheless, data from *in vitro* studies, animal models or patient cohorts have demonstrated a dominant role of SIAH2 as an oncogene in solid tumors and leukemogenesis [12, 19, 28–30]. Our results support this hypothesis, highlighting that the observed increase in SIAH2 expression depends on the histological tumor grade.

The importance of the role of SIAH2 in tumorigenesis has been validated in different investigations that tried to modulate both its abundance and its activity. Schmidt RL and colleagues [31], constructed mutant Siah1/2 proteins with inactivated E3 ligase activity and, therefore, blocked self-ubiquitination and substrate degradation. They found that reduced Siah function increased cell apoptosis and had inhibitory effects on tumor growth, concluding that Siah mediates oncogenic Ras-mediated tumorigenesis in pancreatic cancer. The same group published the first work that analyzed in detail the role of SIAH proteins in lung cancer [32]. The authors demonstrated that SIAH2 plays a critical pathogenic role in human lung cancer and, therefore, opened up a possibility of inhibiting SIAH action as a new anticancer target. Although the conclusions were mainly based on human cell line experiments, a preliminary approach using human lung cancer specimens showed a strong association between increased SIAH protein expression and tumor cell proliferation. However, the major drawback of this study is that if fails to discriminate between the two members of the family, SIAH1 and SIAH2. Furthermore, detailed SIAH2 expression at different levels is lacking.

In another study addressing the role of SIAH1 and SIAH2 in a syngeneic model of breast cancer [33], the authors observed that blockade of SIAH-substrate binding site resulted in reduced tumor growth and angiogenesis. These findings are also consistent with those reported by Qi J and colleagues [34] in a mouse SW1 melanoma model. Inhibition of SIAH2 activity through a RING mutant, dominant-negative form of SIAH2 reduced tumorigenesis, whereas blockade of SIAH2-substrate binding site reduced metastasis but did not have an impact on tumor formation. The same group investigated the contribution of SIAH2 in the TRAMP model of neuroendocrine prostate cancer [19]. Importantly, genetic knockout of Siah2 attenuated the development of neuroendocrine prostate carcinoma. Moreover, both the frequency and size of metastatic lesions were significantly reduced. In line with this observation, in a breast cancer model the authors found that the loss of SIAH2 delayed tumor onset, reduced stromal infiltration, increased tumor perfusion through normalized tumor vasculature, decreased production of proangiogenic growth factors and, ultimately, led to an increased sensitivity to chemotherapy [28].

Recently, Muller and colleagues have demonstrated how SIAH2 accelerates the proteasomal degradation of TYK2, leading to STAT3 inactivation in lung cancer cells [35]. They also showed an inverse correlation between the expression of both proteins in NSCLC samples from different patient cohorts. Moreover, they also found that SCC showed significantly higher levels of SIAH2 compared to ADC. These results are concordant with those obtained in the present work, in which the immunohistochemical analysis showed a higher SIAH2 expression in SCC samples compared to ADC samples. These results highlight not only the importance of SIAH2 expression, but also the expression of some of its substrates that play key roles in the regulation of cancer cell growth and carcinogenesis. We had previously demonstrated a mutual regulation between DYRK2 and SIAH2, by which SIAH2 mediates DYRK2 ubiquitination and controls its expression in response to hypoxia [26]. DYRK2 is a member of an evolutionarily conserved family of dual-specificity tyrosine phosphorylation-regulated kinases (DYRKs) that plays an important role in the DNA damage-signaling pathway and cell proliferation. Although the role of DYRK2 in cancer progression remains unclear, a lower expression in invasive tumors has been described [36]. We demonstrated how the increase in SIAH2 expression consequently suppresses DYRK2 expression. These results suggest the possibility that the accumulation of SIAH2 could be inversely correlated with the expression of key proteins that control tumor progression and invasion, like DYRK2 or STAT3, pointing out SIAH2 as a potential therapeutic target.

For the first time ever, we evaluated SIAH2 expression at different levels in primary lung cancer compared to adjacent normal lung tissue. We found a trend towards an induction of SIAH2 gene expression in SCC compared to adjacent normal lung tissue, although not significant. On the contrary, we did not find differences in SIAH2 gene expression in ADC samples. This is in stark contrast with SIAH2 protein expression studied by western blot, which was significantly increased in both ADC and SCC specimens compared to normal lung tissue. These results clearly point to the possible existence of some alteration at the post-transcriptional level in tumor cells compared to healthy tissue. To date, SIAH2 phosphorylation is possibly the most important control mechanism of SIAH2 self-ubiquitination and degradation [26, 37, 38]. Alterations or changes in the expression of those kinases able to phosphorylate SIAH2 in tumor cells could be one of the underlying mechanisms responsible of this difference between mRNA and protein expression.

They also stand out that the highest SIAH2 protein expression was found in ADC histology compared to SCC histology when analyzed by western blot, since the analysis through immunohistochemistry showed different results. However, these results parallel a previous study that demonstrates a higher expression of SIAH2 in SCC as compared to the ADC analyzed by immunohistochemistry in three different cohorts [35]. Tumor size is a well-known prognostic factor in NSCLC, and is associated with FDG uptake in PET/CT scans. In agreement, we found a strong association between increasing tumor size and ^18^FDG uptake measured as SUV(max), and might be useful for predicting NSCLC patient prognosis. More importantly, SIAH2 protein expression showed a strong correlation with ^18^FDG uptake, which may assist clinicians in stratifying patients at increased overall risk of poor survival.

## Conclusions

Our findings indicate an important association between the expression of SIAH2 and lung cancer, which is also associated with the histological tumor grade, ^18^FDG uptake and with a decrease in the expression of SIAH2 substrates such as DYRK2. These results underline the possible use of SIAH2 as a diagnostic and prognosis marker in lung cancer. Specifically, our findings suggest that the analysis of SIAH2 could improve the diagnosis of lung cancer, and could deepen the current knowledge of its prognosis. Moreover, it could even become a possible therapeutic target for NSCLC.

## Supporting Information

S1 Fig Representative gel of SIAH2 protein expression in lung cancer samples compared to that of the corresponding healthy tissue after normalization to actin signal intensities. The values below the gels indicate SIAH2 and actin protein signal intensities (quantified using ImageJ). SIAH2 relative OD expression in tumor sample compared to normal tissue was calculated after normalization to actin signal intensities.
(TIFF)

S1 Table Clinicopathological characteristics correlated with changes in SIAH2 expression levels (mRNA and protein). (TIFF)

## Abbreviations

SIAH: Seven in absentia homolog
PHDs: Prolyl hydroxylases
DYRK2: Dual-specificity tyrosine-(Y)-phosphorylation regulated kinase 2
SCC: Squamous-cell carcinoma
ADC: Adenocarcinoma
NSCLC: Non-small cell lung cancer
PET/CT scans: Positron emission tomography (*PET*)/ Computed Tomography (CT) scans
^18^FDG: 2-deoxy-2-(^18^F)fluoro-D-glucose
