# Supplementary figures and images for: The Expression of the Ubiquitin Ligase SIAH2 (Seven In Absentia Homolog 2) Is Increased in Human Lung Cancer
Source: PLoS One. 2015 Nov 18;10(11):e0143376. doi: 10.1371/journal.pone.0143376 (PMC4651316; doi:10.1371/journal.pone.0143376)

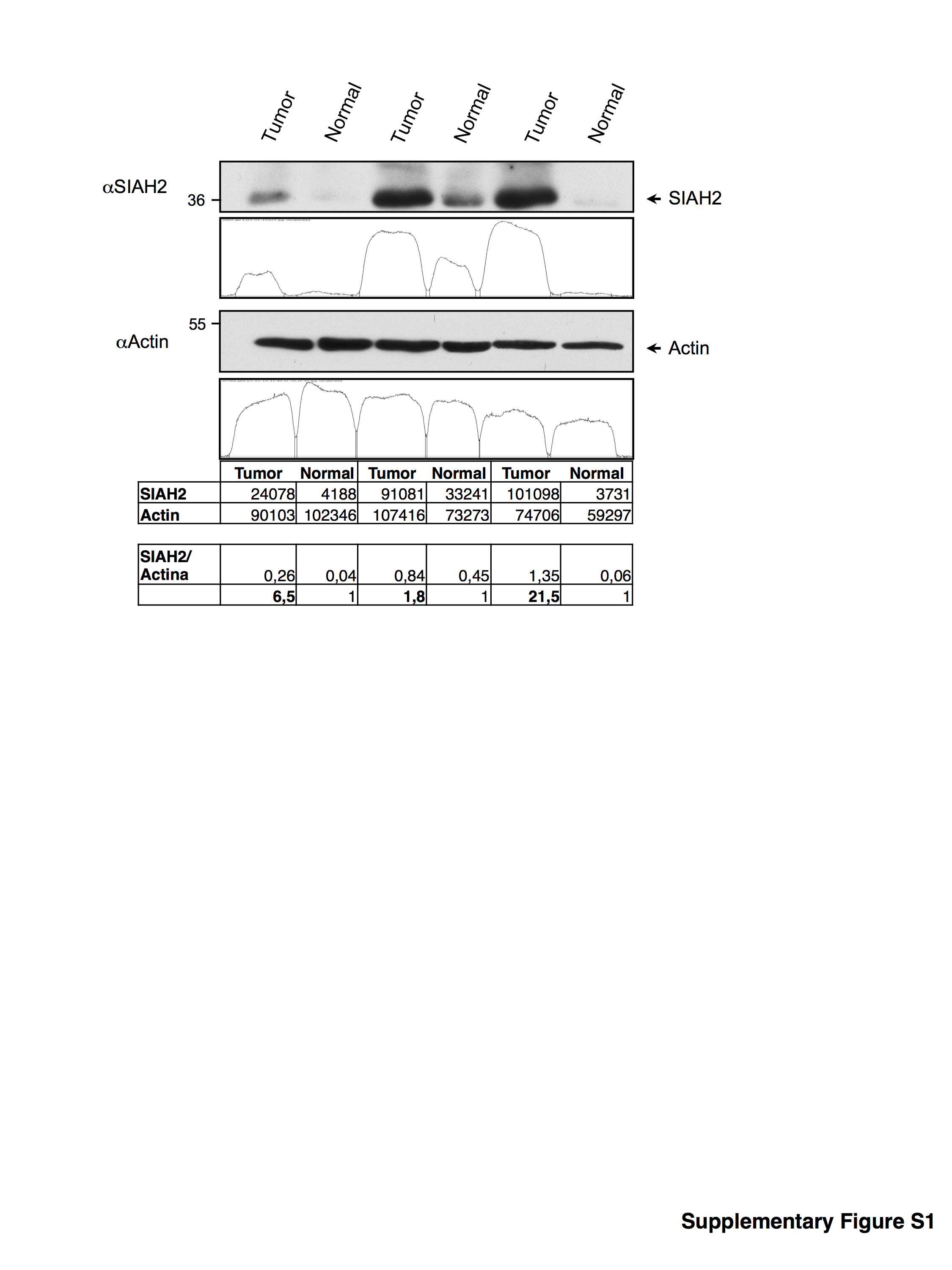

Supplement: S1 Fig — The values below the gels indicate SIAH2 and actin protein signal intensities (quantified using ImageJ). SIAH2 relative OD expression in tumor sample compared to normal tissue was calculated after normalization to actin signal intensities. (TIFF) [file pone.0143376.s001.tiff]

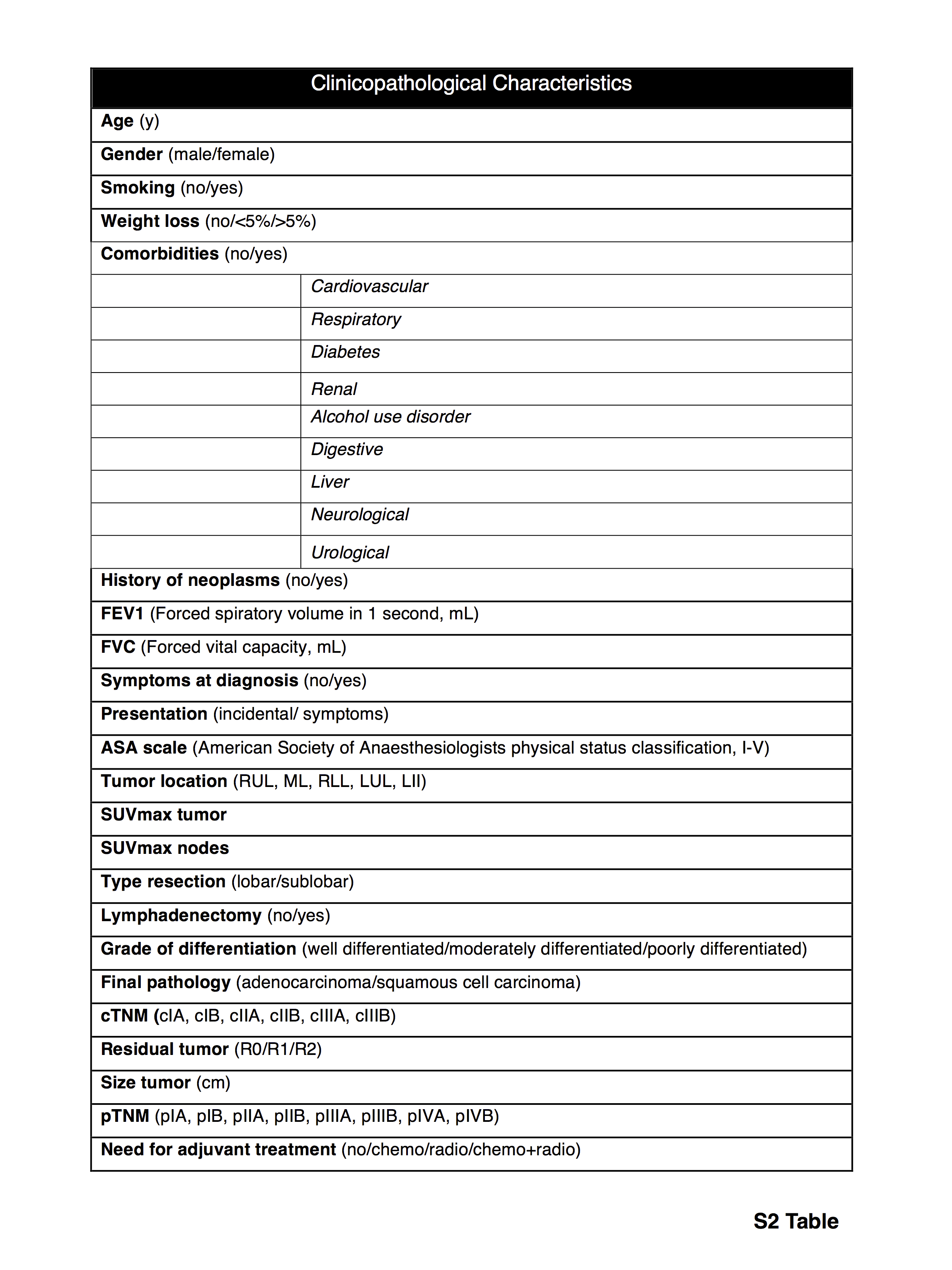

Supplement: S1 Table — (TIFF) [file pone.0143376.s002.tiff]
